# Evaluation and Prediction of the HIV-1 Central Polypurine Tract Influence on Foamy Viral Vectors to Transduce Dividing and Growth-Arrested Cells

**DOI:** 10.1155/2014/487969

**Published:** 2014-06-09

**Authors:** Sergey Shityakov, Carola Förster, Axel Rethwilm, Thomas Dandekar

**Affiliations:** ^1^Department of Anesthesia and Critical Care, University of Würzburg, 97080 Würzburg, Germany; ^2^Department of Virology, University of Würzburg, 97074 Würzburg, Germany; ^3^Department of Bioinformatics, University of Würzburg, 97074 Würzburg, Germany

## Abstract

Retroviral vectors are potent tools for gene delivery and various biomedical applications. To accomplish a gene transfer task successfully, retroviral vectors must effectively transduce diverse cell cultures at different phases of a cell cycle. However, very promising retroviral vectors based on the foamy viral (FV) backbone lack the capacity to efficiently transduce quiescent cells. It is hypothesized that this phenomenon might be explained as the inability of foamy viruses to form a pre-integration complex (PIC) with nuclear import activity in growth-arrested cells, which is the characteristic for lentiviruses (HIV-1). In this process, the HIV-1 central polypurine tract (cPPT) serves as a primer for plus-strand synthesis to produce a “flap” element and is believed to be crucial for the subsequent double-stranded cDNA formation of all retroviral RNA genomes. In this study, the effects of the lentiviral cPPT element on the FV transduction potential in dividing and growth-arrested (G_1_/S phase) adenocarcinomic human alveolar basal epithelial (A549) cells are investigated by experimental and theoretical methods. The results indicated that the HIV-1 cPPT element in a foamy viral vector background will lead to a significant reduction of the FV transduction and viral titre in growth-arrested cells due to the absence of PICs with nuclear import activity.

## 1. Introduction

Retroviral vectors are efficient gene transfer vehicles that deliver transgenes by integration of the viral genome into the genome of host cells. This remarkable ability has been widely exploited for many biomedical applications, including gene therapy. In order to replicate, all retroviruses pursue a very complex process, which is known as reverse transcription. During this event, they transcribe their single-stranded RNA genomes into double-stranded complementary DNA (ds cDNA) prior to its integration into the host genome [1, 2]. The outcome of reverse transcription is a linear DNA with long terminal repeats (LTRs) at the 3′ and 5′ ends of the viral genome. As an example, lentiviruses (HIV-1) have evolved into new forms with a complex reverse transcription strategy including a strand displacement event controlled by the central polypurine tract (cPPT), which serves for plus-strand initiation and priming [3, 4].

**Figure 1:**
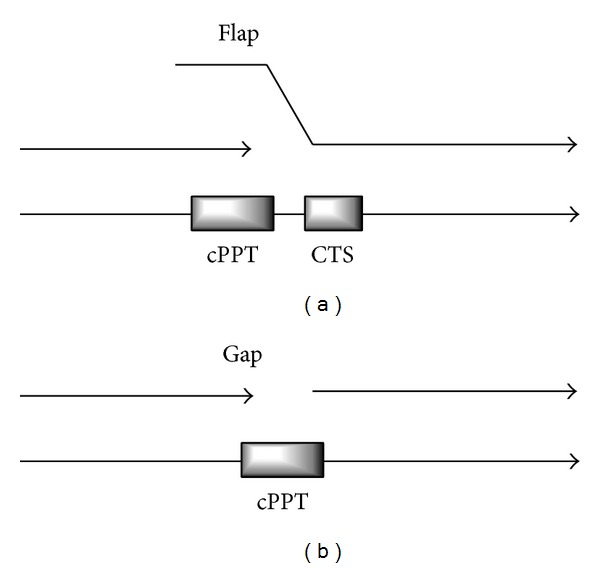
Different reverse transcription outcomes in lenti- (a) and foamy viruses (b). cPPT and CTS abbreviations stand for the central polypurine tract and central termination sequence, respectively.

The HIV-1 reverse transcription produces a linear DNA molecule bearing a “peculiar formation,” known as the central DNA flap element, because the reverse transcription complex stops at the central termination sequence (CTS) (Figure 1(a)). This flap element is believed to be essential for a preintegration complex (PIC) assembly, which is composed of both viral and cellular proteins [5–7] and possess the ability to cross the nuclear pore to enter the nucleus. It was previously reported that some constituents of PIC, such as the viral matrix protein, contained a nuclear localization signal (NLS) for PIC-mediated nuclear import [8, 9]. However, the detailed mechanisms of this process are not yet fully elucidated.

The other retroviruses, such as foamy viruses (FV), have a replication strategy and probably a PIC formation mechanism different from that present in lentiviruses. FVs belong to the Spumavirinae retroviral subfamily and are known as nonpathogenic retroviral species, which are endemic to a number of mammalians such as non-human primates, cats, and cows [10].

The genomic organization of the FVs, including the prototype foamy virus (PFV), which is a molecular cloned simian foamy virus SFVcpz(hu), is similar to that of other complex retroviruses, with several additional open reading frames located in 3′ of the canonical Gag, Pol, and Env genes, including the transcriptional transactivator gene (tas) [11–15].

The foamy viruses have a very broad host range and infect a variety of cell lines, including fibroblastoid, epithelioid, and lymphatic lineages [16]. There are currently a few cell lines that are resistant to FV infection, such as the zebrafish PAC2 and human erythroid precursor cell lines [17]. FV infection leads to massive cell death via apoptosis* in vitro* and without any overt pathogenic effect* in vivo* [18].

Essentially, foamy viruses seem to have diverse outcomes for the plus-strand priming events in contrast to lentiviruses. In FV reverse transcription, the strong stop plus-strand DNA is displaced by DNA elongating from cPPT. This DNA can further be transferred to the 3′ end of the minus-strand DNA or serve as a template for synthesis of a double-stranded LTR. Consequently, cPPT is degraded after completion of the minus-strand cDNA synthesis producing a single-stranded gap (Figure 1(b)) [19–21].

There are a number of FV-based vectors for gene therapy that have been developed [22–24]. These constructs have several desirable properties in comparison to lentiviral vectors. The main characteristics of foamy viral vectors are (i) safety: FVs have not been linked to any established pathology [18, 25]; (ii) wide tissue tropism [10, 26–28]; (iii) viral particles stable and resilient to ultracentrifugation [29]; (iv) packaging efficacy for foreign DNA that is at least 9 kb and offers a valuable tool for the transfer of long genetic elements and therapeutic transgenes [30]; (v) potential for production of high-titre FV vectors suitable for* ex vivo* gene therapy applications [24, 30, 31].

However, these very promising foamy viral vectors have one limitation in their ability to efficiently transduce terminally differentiated and growth-arrested mammalian cells [32] potentially because of their mitosis-dependent integration [22] and the deficiency in forming PICs with nuclear import activity from the absence of the lentivaral cPPT element and central termination sequence.

Therefore, the role of HIV-1 cPPT on FV replication and its mechanism of action is still to be determined. To pursue this goal, the transduction rates of cPPT (HIV-)modified FV vectors in dividing and growth-arrested adenocarcinomic human alveolar basal epithelial (A549) cells were accessed along the investigation of underlying molecular mechanisms and evaluation of their potential for FV-based gene therapy.

## 2. Materials and Methods

**Figure 2:**
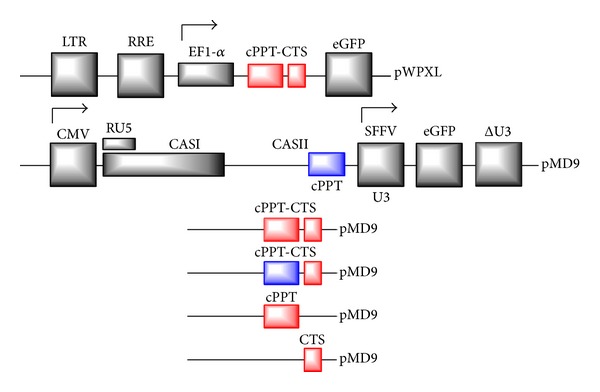
The structural elements of pWPXL and pMD9 parental plasmid backbones are shown: long terminal repeat (LTR) with U3, R, and U5 regions, ref-responsive element (RRE), human elongation factor alpha promoter (EF1-*α*), gene encoding enhanced green fluorescent protein (eGFP), enhancer/promoter of the human cytomegalovirus immediate early gene (CMV),* cis*-acting sequences (CASI and CASII), constitutively active spleen focus forming virus U3 promoter (SFFV U3), and internally deleted U3 region of the 3′ LTR (ΔU3). Foamy viral cPPT was replaced with cPPT (HIV) in different variations. CTS was deleted or inserted in accordance with the scheme shown above.

Recombinant DNA techniques: standard molecular cloning techniques were used for the generation of constructs [33]. A series of plasmid cloning vectors were preceded by the letter “p”. In brief, the pUC19-based intermediate was first generated by ligating a 1.259 kb EcoRI-KpnI fragment from pMD9 (9.940 kb) with a 2.674 kb EcoRI-KpnI fragment from the pUC19 vector to use the proper restriction sites. Then, four pUC19-based plasmids, such as pUC19-cPPT (HIV)-CTS, pUC19-cPPT (PFV)-CTS, pUC19-cPPT (HIV), and pUC19-CTS, were generated by PCR amplification and insertion of the fragments bearing the viral structural elements (Table 1) using relevant oligonucleotides (Table 2) with MfeI-StuI restriction sites. The lentiviral pWPXL vector (10.510 kb) was used as a template for the cPPT (HIV), cPPT (HIV)-CTS, and CTS amplifications. All pMD9-based FV vectors contained the gene encoding enhanced green fluorescent protein (eGFP) under the control of a constitutively active heterologous retroviral U3 promoter to enable the quantification of vector transfer rates (Figure 2).

Cell transfection and purification of supernatant: human embryonic kidney (HEK 293T) cells [34] were seeded in 2 × 10^6^ density into 6 cm dishes a day before the transfection. The cells were transfected with plasmid DNA using a polyethyleneimine transfection reagent (Polysciences Europe GmbH, Eppelheim, Germany) [35]. The PFV transfection mixture contained 2.5 *μ*g of modified pMD9 vectors, 1.25 *μ*g of pCIgag2, 0.5 *μ*g of pCpol-2, and pCenv-1 packaging plasmids [35]. The HIV transfection system contained 2.5 *μ*g of pWPXL, 1.9 *μ*g of psPAX2, and 0.8 *μ*g of pMD2.G (VSV-G) vectors (gifts from Professor Didier Trono), respectively. The pMD9 plasmid without Env (pMD9-w/oEnv) and pcDNA (Invitrogen GmbH, Karlsruhe, Germany) as an empty vector were used as negative controls without a production of viral particles. One day after transfection, the cellular transcription was induced by addition of 10 mM sodium butyrate for 8 hrs [36]. After two days, the supernatant was harvested, passed through a 0.45 *μ*m filter (Merck Millipore, Darmstadt, Germany), and layered onto 6 mL of sucrose cushion (20% in DMEM). The supernatant was centrifuged in a SureSpin 630 rotor (Thermo Scientific, Sorvall, Waltham, MA, USA) at 25000 r.p.m at 4°C for 3 hrs.

**Figure 3:**
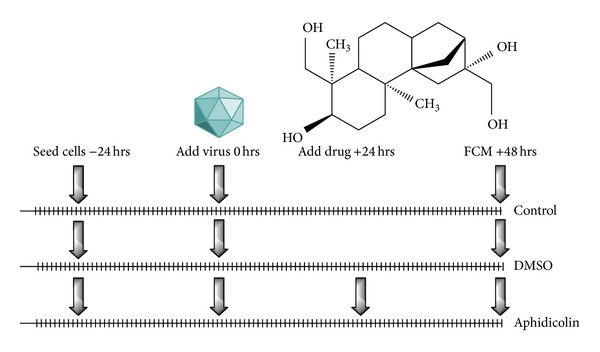
The timescale representation of the transduction experiments with aphidicolin substance to stop a cell cycle in G_1_/S phase. DMSO was added to the A549 cells for a toxicity evaluation and as a solvent for the drug.

Cell transduction and cell cycle experiments: after clarification, the vector-containing supernatants from HEK 293T cells were functionally assayed to transduce the A549 cell-line, which were incubated with them for two days at different conditions. The conditions were created in the experiment to test the generated pMD9-based plasmids to efficiently transduce dividing and nondividing cells (G_1_/S phase of a cell cycle): in Dulbecco's modified Eagles medium (DMEM) as a control, in the presence of dimethyl sulfoxide (DMSO), and in the presence of aphidicolin (Figure 3). The drug was purchased from Sigma-Aldrich (Germany Sigma-Aldrich Chemie GmbH, Germany) as a potent antiviral, antimitotic agent, and DNA polymerase inhibitor, which is used to stop a cell cycle at G_1_/S phase. The drug was dissolved in pure DMSO and added to cells at 5 *μ*g/mL concentration for 24 hrs [32]. The final concentration of DMSO was approximately 0.1% in the medium.

Immunoblotting of viral proteins: analysis of viral protein expression was done essentially as described elsewhere [37]. In brief, the lysates were prepared from the partially purified vector supernatant and from transfected cells by suspension in a detergent-containing buffer. Viral proteins were reacted with anti-Gag [23] and anti-Pol [38] mouse monoclonal antibodies (mAbs) after separation in 8% SDS-PAGE and semidry blotting onto Hybond ECL membranes (Amersham Pharmacia Biotech, Freiburg, Germany). Protein bands were detected by using horseradish peroxidase-coupled secondary antibodies (Dako, Hamburg, Germany) and employing the enhanced chemiluminescence detection system (Amersham Pharmacia Biotech, Freiburg, Germany). The ImageJ software (National Institute of Health, Bethesda, MD, USA) was used to analyse quantitatively the immunoblotting results.

Vector transfer technique: after clarification, the vector-containing supernatants were also assayed functionally by transfer to 1.5 × 10^4^ recipient A549 cells purchased from American Type Culture Collection (ATCC, Manassas, VA, USA). The expression of eGFP per 10^4^ cells was monitored by flow cytometry (FCM) for 48 hrs after the transduction. The vector transfer assays were repeated at least three times.

Viral transduction dynamics and viral titre prediction: the MATLAB R2012a software (MathWorks, Natick, MA, USA) was used to predict the viral transduction dynamics (*f*(*x*)_td_) and viral titre (*f*(*x*)_vt_) for the analysed plasmids. Sigmoid dose-response logistic function was implemented in the MATLAB script M-files using the following equations:

(1) $$\begin{array}{c} f{\left(x\right)}_{\text{td}}=\;\frac{0.01a}{0.01+e^{\left(-bx\right)}}, \end{array}$$

(2) $$\begin{array}{c} f{\left(x\right)}_{\text{vt}}=\;\frac{a}{0.01+e^{x}}, \end{array}$$

where *x* is any independent variable described by linspace function; *a* is a transduction rate (percentage of eGFP cells); *e* is an exponential constant (*e* = 2.718); and *b* integer is a Hill Slope. The linspace function (*x* = linspace(0,48)) was used to generate linearly spaced vectors for 48-hour interval curve. The *b* integer (*b* = 0.21) was adjusted to the *f*(*x*)_td_ logistic function for pWPXL (*y*_1_ = 1./(0.01 + exp⁡(−*x*.∗0.21))) as a reference curve reaching the upper plateau level with maximal effect after 48 hrs (Supplementary Material 1 in Supplementary Material available online at http://dx.doi.org/10.1155/2014/487969). To determine the viral titre dynamics, seven dilutions were inspected in the range from the highest (0.1) to the lowest (10^−7^) concentrations of the virus (*x* = linspace(0,6)).

## 3. Results and Discussion

To be functional, the retroviral system for gene therapy must comprise two principal elements, these being as truncated viral backbones and packaging plasmids. The PFV system therefore includes the Cas I and Cas II (cPPT) backbone elements as a part of pMD9 and pCIgag2, pCpol-2, and pCenv-1 packaging plasmids. On the contrary, the HIV system comprises the *ψ* packaging signal, ref-responsive element (RRE), cPPT-CTS, and posttranscriptional* cis*-acting regulatory element (WPRE) as a part of the pWPXL vector together with psPAX2 (Gag, Pol, rev, and tat) and pMD2.G (VSV-G) packaging vectors to produce G-glycoprotein pseudotyped retroviral particles.

To address the question of the influence of cPPT (HIV) together with the CTS element on the foamy viral cycle, we compared the cell cycle requirements for efficient cell transduction by the pMD9- and pWPXL-based plasmids, expressing the same transgene (eGFP) under control of different promoters (SFFV U3 in pMD6 and EF1-*α* in pWPXL) in dividing and growth-arrested (G_1_/S) A549 cells. Although there are the previously published reports that have already focused on the cell cycle aspects of retroviral infection [39, 40], the investigation is the first direct attempt to assess the role of cPPT modifications for the two genera of viruses: lentiviruses (HIV-1) and spumaviruses (PFV).

**Figure 4:**
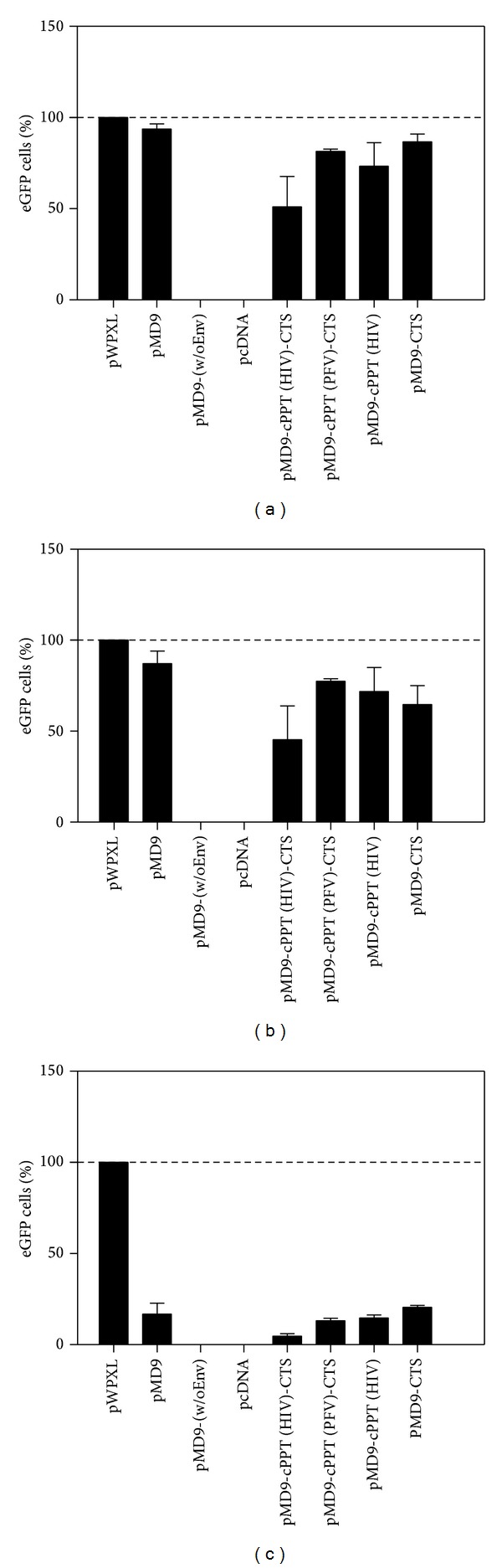
The summarized statistics of retroviral replication efficiency (transduction rate) in the A549 cells. The pMD9-based plasmids were tested to efficiently transduce dividing and growth-arrested cells (G_1_/S phase of cell cycle). Three conditions were analysed in the experiment: control (a); DMSO (b); aphidicolin (c). The mean ± SD values from three independent assays are shown.

The analysis of the viral transduction rates revealed that both untreated or DMSO-treated cells experienced minor decreases in transduction rates for the pMD9-based plasmids with different cPPT modifications in comparison to pMD9 and pWPXL controls (Figures 4(a) and 4(b)). These results indicated that the productive infection was observed only if target cells were allowed to pass through mitosis. On the other hand, there was a huge difference in the relative transduction efficiencies of the vectors when the cells were G_1_/S-arrested for 24 hours after transduction; while the efficiency of pWPXL was approximately twenty- (pMD9-cPPT (HIV)-CTS) to fivefold (pMD9-CTS) higher than that of the pMD9-based vectors (Figure 4(c)). The short inserts, such as CTS and cPPT (HIV), also displayed an insignificant reduction in the transduction rates observed in dividing cells. The longer insert (cPPT (HIV)-CTS) resulted in an almost 50% reduction in transduction rates compared to the parental vector controls (Figures 4(a) and 4(b)). One point to note is that the different promoters directed the eGFP transgene expression in the vectors. In this regard, it is always inaccurate to compare the levels of transgene expression under control of the different promoters (SFFV U3 versus EF1-*α*).

**Figure 5:**
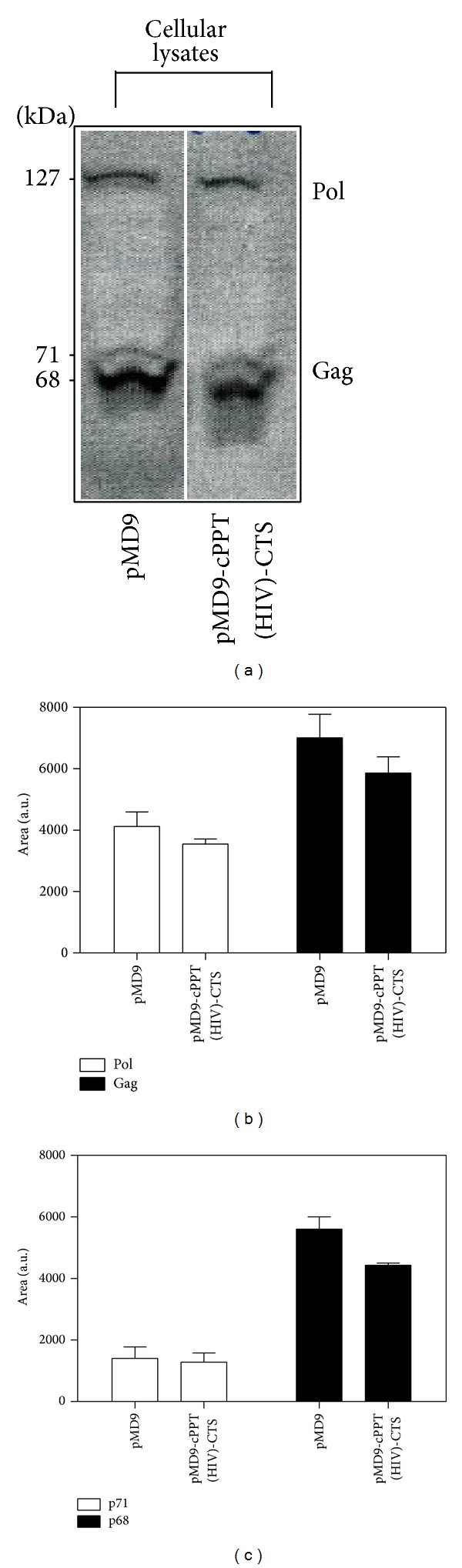
Analysis of pMD9 parental vector and its modification (pMD9-cPPT (HIV)-CTS). Detection of PFV proteins in HEK 293T cells (a) contransfected with the retroviral vectors was measured quantitatively for Gag/Pol (b) and Gag fractions (p71, p68) (c). The mean ± SD values from three independent assays are shown. The area is measured in arbitrary units, which are abbreviated as a.u.

**Figure 6:**
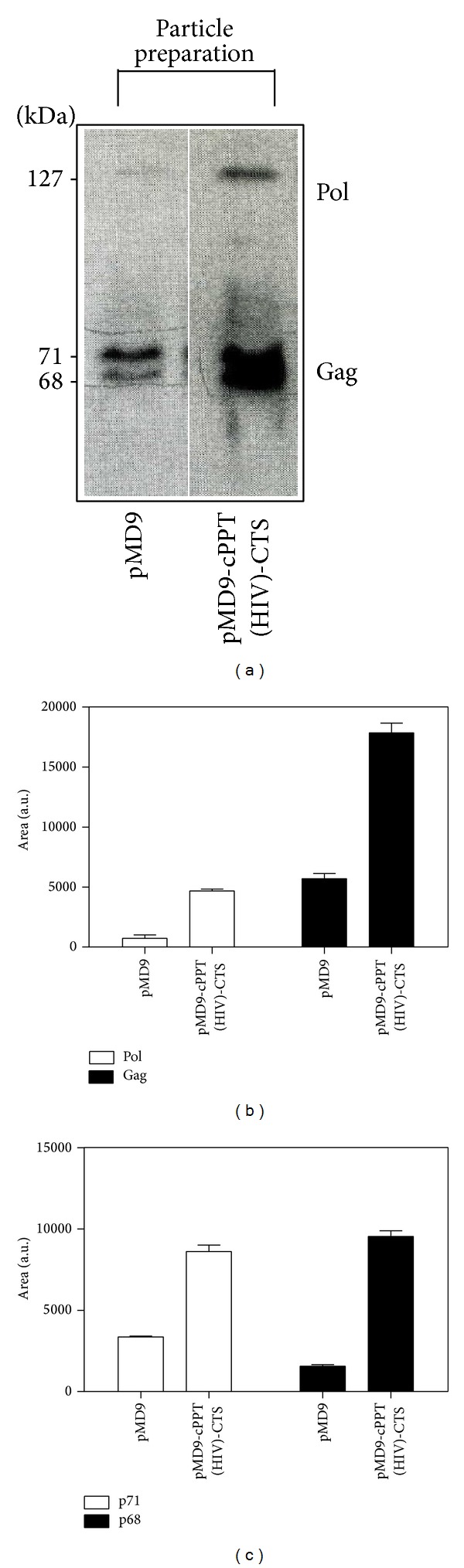
Analysis of pMD9 parental vector and its modification (pMD9-cPPT (HIV)-CTS). Detection of PFV proteins in partially purified viral particles produced by HEK 293T cells (a) contransfected with the retroviral vectors was measured quantitatively for Gag/Pol (b) and Gag fractions (p71, p68) (c). The mean ± SD values from three independent assays are shown. The area is measured in arbitrary units, which are abbreviated as a.u.

FV replication strategy differs in many aspects from that of orthoretroviruses with the structural proteins displaying many unique functions not found for the corresponding orthoretroviral proteins. The FV structural proteins, Gag, Prt-RT, and Env are initially translated in polyprotein forms that are subsequently cleaved by cellular proteases (Gag and Prt-RT proteins), while Env polyprotein is cleaved by viral protease [15]. Interestingly, the protein analysis of the pMD9-cPPT (HIV)-CTS plasmid compared to its parental form indicated that the A549 transduction for the modified vector was reduced when judged by the Gag/Pol decrease in the HEK 293T cellular lysates (Figures 5(a) and 5(b)). However, this was offset by a significant increase in the capacity to encapsidate Gag and Pol, producing a large pool of defective virus-like particles (Figures 6(a) and 6(b)).

The organization of Gag matrix protein into MA (p17 matrix), CA, (capsid p24), and NC (p6, nucleocapsid) with distinct cleavage sites observed in HIV-1 is absent in FVs. The only processing of FV Gag observed in the course of FV particle morphogenesis* in vivo* occurs at the C terminus of the molecule, removing a 3 kDa peptide (p3) and producing a shortened protein of 68 kDa. However, three internal secondary cleavage sites have been characterized* in vitro* that seem to be important during steps of the FV replication cycle upon entry into target cells [15]. Thereby, in the 293T cellular lysates, the domination of the cleaved versions of Gag (p68) for either the pMD9 or pMD9-cPPT (HIV)-CTS constructs (Figure 5(c)) was observed. However, the protein analysis of the particle preparation revealed the prevalence of an uncleaved Gag fraction (p71) for pMD9, indicating the incomplete proteolytic cleavage of the FV capsid protein. The same fractions were detected in equal amounts in partially purified particles produced by HEK 293T cells and cotransfected with the modified plasmid (Figure 6(c)).

**Figure 7:**
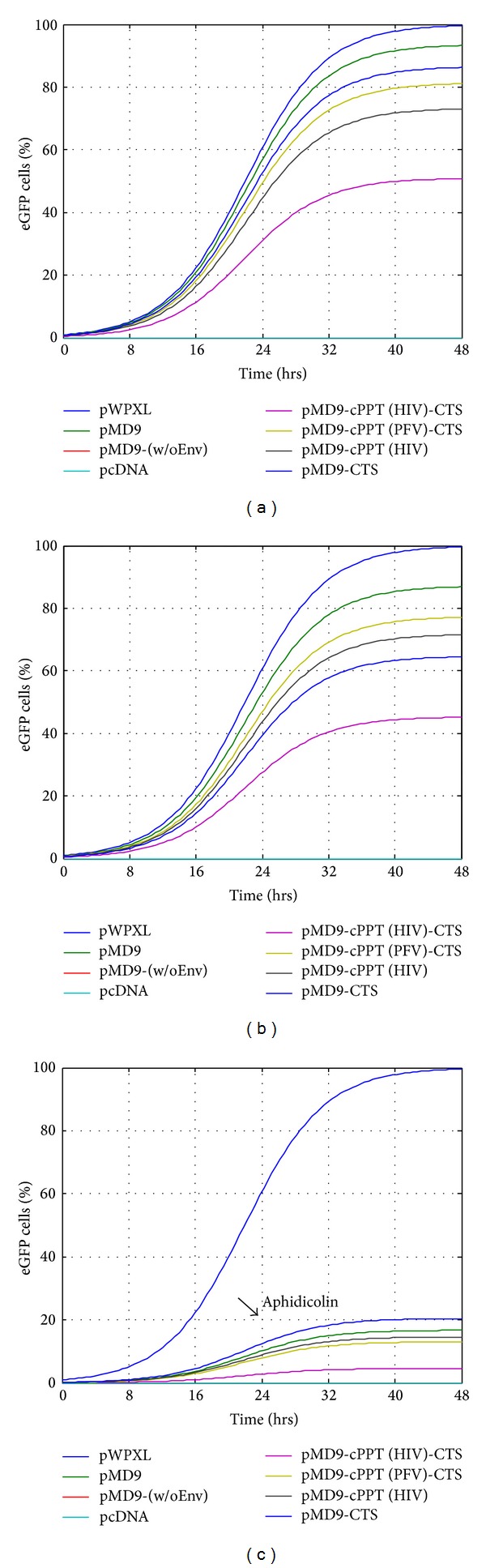
Dynamic *f*(*x*)_td_ function: logistic dose-response curve to predict and model viral infection cycle of retroviral replication efficiency in the A549 cells. The pMD9-based plasmids were tested to efficiently transduce dividing and growth-arrested cells (G_1_/S phase of cell cycle). Three conditions were analysed in the experiment: control (a); DMSO (b); aphidicolin (c).

**Figure 8:**
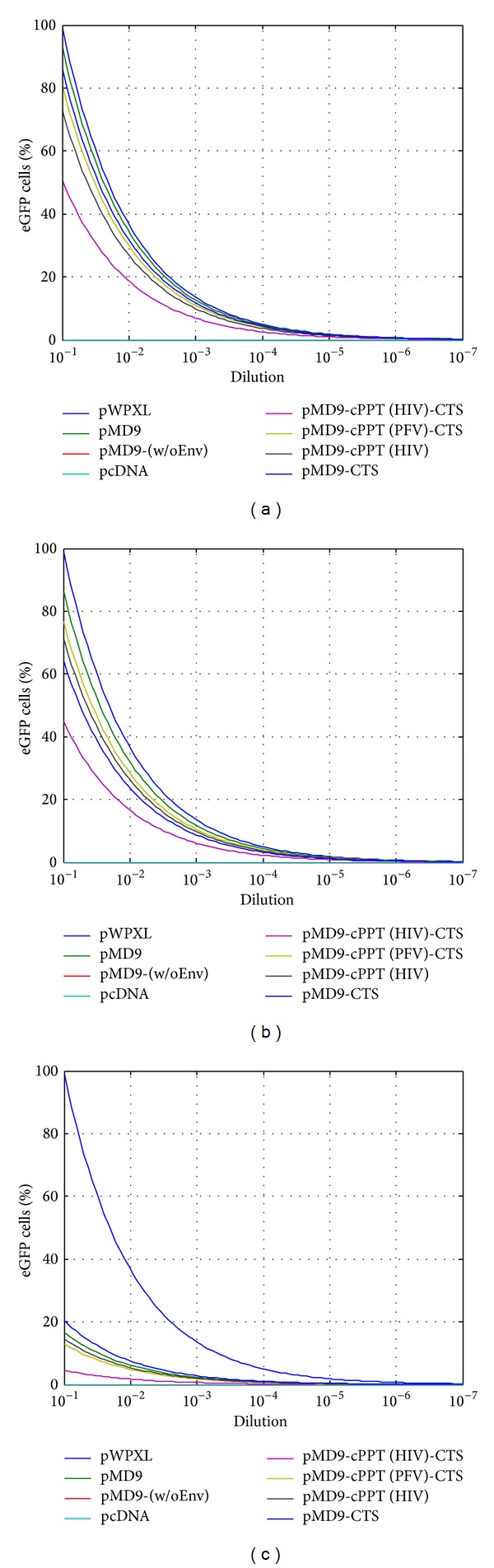
Dynamic *f*(*x*)_vt_ function: logistic dose-response curve to predict and model the viral titre. The pMD9-based vectors were tested to efficiently transduce dividing and growth-arrested cells (G_1_/S phase of cell cycle). Three conditions were analysed in the experiment: control (a); DMSO (b); aphidicolin (c).

To assess the viral transduction dynamics and viral titre, the logistic dose-response function was transformed according to (1) and (2), to model the experimentally determined efficiency of the retroviral replication as percentage of eGFP cells observed in the A549 cells. Both parameters demonstrated a correlation to the aforementioned data shown in Figure 4 indicating some decrease of *f*(*x*)_td_ and *f*(*x*)_vt_ in dividing cells (Figures 7(a), 7(b), 8(a), and 8(b)). Similarly, the FV inability to transduce at a significant extent the growth-arrested cells provided very low *f*(*x*)_td_ and *f*(*x*)_vt_ values (Figures 7(c) and 8(c)).

Overall, the results might be in conflict with a published report claiming that simian FV vectors can efficiently transduce aphidicolin-treated cells [40]. While the results cannot rule out strain-specific differences in the vector systems used, it is highly likely that simian FV vectors require mitosis for their efficient transduction. Moreover, they require a breakdown of the nuclear envelope for a successful nuclear entry [39, 41]. Lentiviral vectors also enter the nucleus using specific nuclear localization signals [8, 42].

It is more complicated that after viral disassembly in a cytoplasm some retroviral NLS sequences are recognized by host-cell import factors such as transportins that mediate nuclear targeting of PICs via the nuclear pore complex [7]. Several similar sequences are present in HIV-1, most significantly in the virus integrase [8, 43]. Foamy viral Gag and Pol proteins also include NLS sequences [38], but they were recently shown to be nonfunctional [44].

Like HIV-1, PIC formation is also present in FVs before the transport of double-stranded cDNA of the virus to the cellular chromatin. Therefore, it is an essential part of replication of FVs [45]. However, the lower transduction efficiency of FV vectors in growth-arrested cells, in comparison with lentiviral forms, could be due to the fact that the foamy viral NLSs are being partially occluded in PIC [46], showing that PFV generates 2-LTR circles in nondividing cells. However, foamy virus vector preparations already contain 2-LTR circles, so their presence is not useful for monitoring PFV DNA entry into the nucleus although it is still possible that there are further functional blocks for successful PFV integration into a host genome after the nuclear translocation of its DNA.

In addition, some retroviruses (HIV-1) have a tendency to package some cellular proteins nonspecifically, and DNA ligase activity has been previously demonstrated for viral particles [47]. Therefore, these steps might also take place in intracellular or extracellular FV particles that contain cDNA molecules. Some studies even detected additional FV LTR circles by PCR in aphidicolin-arrested but not serum-deprived cultures [46]. There is a possibility that FV vectors use distinct mechanisms for nuclear import, as both the viral genome and Gag proteins accumulate near the centrosome/MTOC and wait for cell division. They then disassemble, releasing PIC [46].

The presence of cPPT, that serves as a primer for plus-strand synthesis to produce the HIV-1 “flap” element, has not been reported so far for FVs. But plus-strand synthesis that ultimately leads to the double-stranded cDNA, the molecular moiety that actually integrates into the host chromatin is a universal feature of all retroviruses including spumaviruses, the subfamily of FVs. The mechanism of formation of ds cDNA in FV might have a different mechanism.

Taken all together, the cell cycle dependences of foamy virus infection have demonstrated that vectors based on these viral genomes are not sufficient for specific targeting of nondividing cells. Nonetheless, the neurotropism of foamy viruses is worth testing in animal models. Further studies will be needed to directly compare foamy viral and lentiviral vectors in preclinical gene therapy experiments and define the nature of the stable FV vector transduction intermediate in quiescent cells.

## 4. Conclusion

In the experiments, modified foamy viral vectors showed a reduction in transduction rates of dividing, especially G_1_/S-arrested A549 cells in comparison to lentiviral forms. The findings have confirmed that mitosis is a critical phase in the cell cycle for FV transduction, which was previously observed by Bieniasz and coauthors as an absence of the FV protein expression in G_1_/S- and G_2_-arrested cells [22].

From the data, it is clear that foamy viruses are not able to infect nondividing cells efficiently most likely because of lower integration efficiency compared to lentiviruses unless cells are undergoing mitosis. The results are in accordance with the previous findings of Trobridge and Russell but are contrary to the observations published by Mergia and coauthors [32, 40]. Hence, the current study leads to the fact that foamy viral vectors could be further improved to be effective in gene therapy to target nondividing cells.

It is shown how foamy viral vectors can infect the A549 cells in dividing and growth-arrested states; cell-type specific experiments and molecular cloning strategies were included to clearly demonstrate that HIV-1 cPPT cannot be substituted for PFV cPPT without loss of functionality. This might be because the cognate reverse transcription enzyme is required for recognition of cPPT and CTS sequences. Furthermore, validated simulation data and curves were provided for the dynamic FV replication potential.

## Supplementary Material

Sigmoid dose-response logistic function, predicting the viral transduction dynamics (f(x)_td_) and viral titre (f(x)_vt_), was implemented in the MATLAB script M-files using the following equations:
f(x)_td_= 0.01a/(0.01+e(^-bx^)
f(x)_vt_= a/0.01+e^x^
where *x* is any independent variable described by *linspace* function; *a* is a transduction rate (percentage of eGFP cells); *e* is an exponential constant (*e* = 2.718); and *b* integer is a Hill Slope. The *linspace* function (*x* = *linspace* (0, 48)) was used to generate linearly spaced vectors for 48-hour interval curve. The *b* integer (*b* = 0.21) was adjusted to the f(x)_td_ logistic function for pWPXL (y1=1.0./(0.01+exp(-x.∗0.21)) as a reference curve reaching the upper plateau level with maximal effect after 48 hrs. To determine the viral titre dynamics, seven dilutions were inspected in the range from the highest (0.1) to the lowest (10^−7^) concentrations of the virus (*x* = *linspace* (0, 6)).

## Figures and Tables

**Table 1:** Sequences of the viral structural elements used in the construction of modified expression vectors.

Element	Length (bp)	Sequence
cPPT (PFV)	9	AGGAGAGGG
cPPT (HIV)	33	ATCCACAATTTTAAAAGAAAAGGGGGGATTGGG
CTS	16	AAAAATTCAAAATTTT

**Table 2:** Forward and reverse primers for a PCR amplification of the fragments used in the construction of modified expression vectors.

Fragments	Primers
cPPT (PFV)-CTS	5′-TATACAATTGCAGGAGAGGGATTGGGGGGTACAGTGCAG-3′
	5′-TATAAGGCCTCTGTCCCTGTAATAAACC-3′

cPPT (HIV)-CTS	5′-TATACAATTGATGGCAGTATCCAC-3′
	5′-TATAAGGCCTCTGTCCCTGTAATAAACC-3′

cPPT (HIV)	5′-TATACAATTGATGGCAGTATCCAC-3′
	5′-TATAAGGCCTGTAATTTGTTTTTGTAATTCT-3′

CTS	5′-TATATACAATTGGGGGGTACAGTGCAGGGG-3′
	5′-TATATAAGGCCTTCCCTGTAAACCCGAAAATTTTG-3′
