# Supplementary material for: Evaluation and Prediction of the HIV-1 Central Polypurine Tract Influence on Foamy Viral Vectors to Transduce Dividing and Growth-Arrested Cells
Source: ScientificWorldJournal. 2014 Jun 9;2014:487969. doi: 10.1155/2014/487969 (PMC4070436; doi:10.1155/2014/487969)
Supplement: Supplementary file 1 — Sigmoid dose-response logistic function, predicting the viral transduction dynamics (f(x)td) and viral titre (f(x)vt), was implemented in the MATLAB script M-files using the following equations: f(x)td= 0.01a/(0.01+e(-bx) f(x)vt= a/0.01+ex where x is any independent variable described by linspace function; a is a transduction rate (percentage of eGFP cells); e is an exponential constant (e = 2.718); and b integer is a Hill Slope. The linspace function (x = linspace (0, 48)) was used to generate linearly spaced vectors for 48-hour interval curve. The b integer (b = 0.21) was adjusted to the f(x)td logistic function for pWPXL (y1=1.0./(0.01+exp(-x.∗0.21)) as a reference curve reaching the upper plateau level with maximal effect after 48 hrs. To determine the viral titre dynamics, seven dilutions were inspected in the range from the highest (0.1) to the lowest (10−7) concentrations of the virus (x = linspace (0, 6)). [file 487969.f1.docx]

**Supplementary material 1**

**Schemes of plasmids used in the study**
